# Immediate and long-term effects of BCI-based rehabilitation of the upper extremity after stroke: a systematic review and meta-analysis

**DOI:** 10.1186/s12984-020-00686-2

**Published:** 2020-04-25

**Authors:** Zhongfei Bai, Kenneth N. K. Fong, Jack Jiaqi Zhang, Josephine Chan, K. H. Ting

**Affiliations:** 1 Department of Rehabilitation Sciences, The Hong Kong Polytechnic University, Kowloon, Hong Kong SAR; 2 Department of Occupational Therapy, Shanghai YangZhi Rehabilitation Hospital (Shanghai Sunshine Rehabilitation Center), Shanghai, China; 3 Department of Rehabilitation Sciences, Tongji University School of Medicine, Shanghai, China; 4 School of Occupational Therapy, Institute of Health Sciences, Texas Woman’s University, Houston Center, USA; 5 University Research Facility in Behavioral and Systems Neuroscience, The Hong Kong Polytechnic University, Kowloon, Hong Kong SAR

**Keywords:** Brain-computer interface, Stroke, Hemiparetic upper extremity function, Motor imagery, Movement attempt, Neural mechanism

## Abstract

**Background:**

A substantial number of clinical studies have demonstrated the functional recovery induced by the use of brain-computer interface (BCI) technology in patients after stroke. The objective of this review is to evaluate the effect sizes of clinical studies investigating the use of BCIs in restoring upper extremity function after stroke and the potentiating effect of transcranial direct current stimulation (tDCS) on BCI training for motor recovery.

**Methods:**

The databases (PubMed, Medline, EMBASE, CINAHL, CENTRAL, PsycINFO, and PEDro) were systematically searched for eligible single-group or clinical controlled studies regarding the effects of BCIs in hemiparetic upper extremity recovery after stroke. Single-group studies were qualitatively described, but only controlled-trial studies were included in the meta-analysis. The PEDro scale was used to assess the methodological quality of the controlled studies. A meta-analysis of upper extremity function was performed by pooling the standardized mean difference (SMD). Subgroup meta-analyses regarding the use of external devices in combination with the application of BCIs were also carried out. We summarized the neural mechanism of the use of BCIs on stroke.

**Results:**

A total of 1015 records were screened. Eighteen single-group studies and 15 controlled studies were included. The studies showed that BCIs seem to be safe for patients with stroke. The single-group studies consistently showed a trend that suggested BCIs were effective in improving upper extremity function. The meta-analysis (of 12 studies) showed a medium effect size favoring BCIs for improving upper extremity function after intervention (SMD = 0.42; 95% CI = 0.18–0.66; *I*^*2*^ = 48%; *P* < 0.001; fixed-effects model), while the long-term effect (five studies) was not significant (SMD = 0.12; 95% CI = − 0.28 – 0.52; *I*^2^ = 0%; *P* = 0.540; fixed-effects model). A subgroup meta-analysis indicated that using functional electrical stimulation as the external device in BCI training was more effective than using other devices (*P* = 0.010). Using movement attempts as the trigger task in BCI training appears to be more effective than using motor imagery (*P* = 0.070). The use of tDCS (two studies) could not further facilitate the effects of BCI training to restore upper extremity motor function (SMD = − 0.30; 95% CI = − 0.96 – 0.36; *I*^2^ = 0%; *P* = 0.370; fixed-effects model).

**Conclusion:**

The use of BCIs has significant immediate effects on the improvement of hemiparetic upper extremity function in patients after stroke, but the limited number of studies does not support its long-term effects. BCIs combined with functional electrical stimulation may be a better combination for functional recovery than other kinds of neural feedback. The mechanism for functional recovery may be attributed to the activation of the ipsilesional premotor and sensorimotor cortical network.

## Background

Motor deficit is the most common sequela after stroke, resulting in severe negative impacts on activities of daily living and social participation for patients [1]. Spontaneous recovery usually occurs within the first 3 months after the onset of stroke; however, there exists a great deal of variability in recovery across patients, particularly patients with severe deficits, who tend to recover less and more slowly [2]. With regard to the importance of motor training in facilitating motor recovery after stroke, various rehabilitation training protocols, such as task-specific training and constrained-induced motor training have been applied in regard to stroke [3, 4]. However, these protocols are limited in patients with severe motor function deficit, due to the voluntary participation of hemiparetic hands. On the other hand, brain-computer interface (BCI) technology does not involve the direct volitional control of hemiparetic hands in training; therefore, it may be promising for these patients.

The term “BCIs” refers to systems that capture the features of brain activity and translate them into computerized commands to control external devices, which can be communication devices [5], functional electrical stimulation (FES) [6], or exoskeleton robots [7], among others. To acquire brain activity signals, either invasive or non-invasive strategies can be used. Invasive BCIs can acquire spatiotemporal signals and have a great capacity to distinguish more dimensions of patients’ intent through implants in the brain cortex [8]. However, non-invasive BCIs, using signals collected from electroencephalography (EEG), magnetoencephalography (MEG), functional near-infrared spectroscopy (fNIRS), or functional magnetic resonance imaging (fMRI), may be more promising than the invasive strategy in reality, due to safety and ethical issues [9]. Among them, the EEG signal-based BCI is the most commonly used system because of its relatively simple and inexpensive equipment requirements, as well as rich sources regarding its temporal resolution (e.g., visually evoked potential, P300, slow cortical potential) and frequency (e.g., power in given frequency bands) domains, the information can be extracted as the feature for controlling external devices [10]. The EEG signal-based BCI captures the signal of the event-related and time-locked decrease or increase in the oscillatory power in given frequency bands; in other words, the event-related desynchronization (ERD) or event-related synchronization (ERS), respectively [11, 12]. At present, hybrid BCI systems that combine more than one signal can provide more efficient natural control of external devices [13].

In 2009, Daly et al. [14] reported the first case study concerning the feasibility of an EEG signal-based BCI combined with FES in regard to stroke rehabilitation. After a three-week training period, the patient under study regained volitional isolated index finger extension, suggesting the potential immediate effects of this method on motor recovery [14]. In subsequent well-designed studies, the immediate effects of BCIs on motor function were confirmed [15, 16] and researchers also explored the immediate effects on improvements in spasticity [15], muscle strength [16], and activities of daily living [16, 17]. However, many well-known rehabilitation strategies, such as virtual reality [18] and mirror therapy [19], which showed superior immediate effects, might not have long-term effects across time. The latest meta-analysis summarized the immediate clinical effects of BCIs based on nine studies; the overall results support the effectiveness of BCI training on the improvement of upper extremity motor function in stroke [20]. However, the evidence related to the immediate effects of BCIs in other aspects (e.g., spasticity, strength, etc.) and corresponding long-term effects were not certain.

At present, brain activity during motor imagery (MI) and movement attempts can be used to trigger external devices. However, it is believed that these two mental tasks have different mechanisms in regard to promoting neural plasticity. MI is a mental rehearsal of movements without any real movement. The neural substrates of MI have been extensively studied with neuroimaging techniques and have been found to possess substantial overlapping with the neural network of motor execution, such as in the contralateral supplementary motor area (SMA), contralateral postcentral gyrus, contralateral superior parietal lobe, and ipsilateral prefrontal cortex [21, 22]. On the other hand, it is well known that the mu (8–13 Hz) and beta (13–30 Hz) rhythms over the primary motor cortex (M1) and bilaterally across the precentral motor cortex desynchronize during motor execution, movement attempts, and MI [23, 24]. A study using electrocorticography shows that both motor execution and MI induced ERD in mu and beta bands accompanied by ERS at high frequencies (76–100 Hz) over contralateral M1, but the former had larger changes than the latter [8]. Transcranial magnetic stimulation (TMS) further proved the enhanced cortical excitability of M1 during MI, as measured by increased motor-evoked potential (MEP) [25]. In 2010, Prasad et al. reported on the use of an MI-based BCI system in regard to five patients with chronic stroke; their results show the proof-of-concept of BCI training in regard to improving motor function [26].

In addition to MI, movement attempts (i.e., patients attempt to move their paretic hands, even though they have completely lost voluntary movements) have been proposed for BCIs in stroke [14]. A previous neuroimaging study indicated that the cortical activity of movement attempts closely followed the somatotopic organization of motor execution in patients after spinal cord injuries [27]. The neural mechanism of movement attempt-based BCIs refers to Hebbian plasticity, which is different from that of MI. Hebbian plasticity explains a form of enhanced synaptic plasticity if a close timing order of pre- and post-synaptic activity occurs [28]. Post-synaptic spiking after presynaptic firing can result in short-term potentiation, which is largely dependent on the N-methyl-D-aspartate receptor [29]; the sensorimotor loop is disrupted in patients with stroke due to the loss of voluntary movements, but the capacity of motor planning may still be retained. A previous study indicated that movement attempts could be extracted from EEGs for patients with complete hand paralysis [30] and can be used to trigger external devices (e.g., robot arms), potentially restoring the normal timing order of motor preparation, execution, and peripheral muscle effectors [30]. Therefore, through this form of BCI training, patients could learn to control the brain oscillatory activity induced by movement attempts through immediate and correct somatosensory feedback, and a new sensorimotor loop could be established [15, 16]. Recently, researchers have argued that movement attempts are more informative than MI, because patients have to actively suppress the movement of extremities in MI, while it is more natural to attempt movement [31].

To establish a closed sensorimotor loop, BCIs are combined with different external devices to achieve feedback regarding self-regulated brain activity. FES has been used in BCI systems to elicit muscle contraction in the paretic arm, by delivering electrical stimulation [32]. It has been proven that FES is able to facilitate the efficacy of closed sensorimotor loop during BCI training, by increasing the patient’s movement awareness during motor training and by enhancing corticospinal excitability [33]. Robots (e.g., exoskeletons and orthosis) have also been integrated in BCI systems to provide proprioceptive feedback. The clinical effects of robot-assisted therapy were found to be modest in comparison with conventional rehabilitation, according to the results of a large-scale study [34]. However, when integrated with BCI training, patients can control their movements with the assistance of robotic devices more voluntarily, thus improving their participation [15]. In addition, visual feedback is used in BCI training to provide simple and fast feedback regarding brain activity [35]. As indicated in the review conducted by van Dokkum et al. [36], different external devices appear to play different roles in the closed sensorimotor loop. For instance, BCIs combined with FES can link movement intention with muscle contraction, turning the bottom-up approach of FES into a top-down approach. Moreover, a study carried out by Ono et al. [37] indicated that the external device providing proprioceptive feedback tended to be more effective than visual feedback in clinical outcomes, suggesting that external devices may significantly boost the effects of BCIs. To the best of our knowledge, there have been no studies directly comparing the effects of different external devices combined with BCI training in clinical outcomes.

Anodal stimulation of transcranial direct current stimulation (tDCS), is capable of exciting the cortex [38]. Recent studies have found it effective in increasing the ERD of mu rhythm during MI [39], and thereby improved motor performance when combined with BCI training based on MI tasks [40]. Although the clinical effects of BCIs in stroke can be potentiated by a preceding tDCS to the cortex, the effects of tDCS in facilitating BCI applications, in regard to restoring motor function for stroke, have not been reviewed before.

A recent meta-analysis by Cervera et al. [20] evaluated the immediate effects of BCIs on the improvement of upper extremity motor function for stroke. The current study aims: (1) to investigate both the immediate and long-term clinical effects of BCI training on the improvement of hemiparetic upper extremity function, and the related neural plasticity changes elicited by BCIs in patients after stroke; (2) to study the potential differences in treatment effects caused by different training paradigms for BCIs measuring signals from the motor cortex (e.g., MI-based BCIs and movement attempt-based BCIs); (3) to explore the potential differential effects of BCIs when combined with different kinds of external devices; and (4) to explore the potentiating effect of tDCS on BCI training.

## Methods

The current systematic review and meta-analysis is reported in accordance with the preferred reporting items for systematic reviews and meta-analyses statement [41].

### Search strategy

A systematic computerized literature search was conducted by one of the authors (ZB) across the following databases: PubMed, Medline, EMBASE, CINAHL, CENTRAL, PsycINFO, and PEDro. In each database, the search was conducted using a combination of keywords “stroke OR cerebral infarction OR cerebral hemorrhage OR cerebral vascular accident AND brain-machine interface OR brain-computer interface”. A manual search was also conducted, which included screening the reference lists of previous systematic reviews and searching Google Scholar using the same keywords. The published data were not limited and the last search took place on August 1st, 2019.

### Selection criteria

The following criteria were applied in the article selection. Studies were included if they met all of the following inclusion criteria. 1) Either single-group studies or controlled studies. The control intervention could be sham BCI training or conventional training without BCIs. 2) Aimed to evaluate the effects of BCIs on hemiparetic upper extremity functional recovery. 3) The BCI training was administered across more than one session. 4) Subjects were adults with stroke. 5) At least one assessment related to clinical effects was conducted before and after the intervention. 6) Peer-reviewed journal articles or conference proceedings with full texts. 7) Published in English. Studies were excluded if they met one of the following exclusion criteria. 1) Studies involving subjects with brainstem stroke, lock-in syndrome, or traumatic brain injuries. 2) Studies only concerning brain signal detection and decoding. 3) Studies that published updated data. First, two reviewers (ZFB and JQZ) independently screened all of the records based on the titles and abstracts. Second, the remaining records were imported into Endnote X8 and the full texts were downloaded. The two reviewers read the full texts in order to decide which studies met our criteria. Then, a face-to-face discussion took place to reach an agreement regarding study inclusion. When necessary, a third reviewer (KNKF) joined the discussion and resolved any discrepancies.

### Data extraction

Two reviewers independently conducted data extraction. A customized form was pre-produced for data extraction regarding the included studies’ general characteristics and results. The general characteristics extracted consisted of authors, year of publication, study design, sample size, age of subjects, average time since stroke, interventions, brain signals, and the dosage of the interventions. Information related to both clinical effects and neural mechanisms were extracted. For the clinical effects reported in controlled studies, mean scores and standard deviations (SDs) of the outcomes before and after the interventions were extracted, as well as the mean change scores and SDs for meta-analyses. If the data reported in articles could not be used for data pooling, the authors of the articles were contacted to request the necessary data. After the independent data extraction, the two reviewers again held a face-to-face discussion to reach an agreement regarding data extraction. When necessary, a third reviewer (KNKF) joined the discussion and resolved any discrepancies.

### Methodological quality assessment

Two independent reviewers critically appraised the methodological quality of the controlled studies using the Physiotherapy Evidence Database (PEDro) rating scale [42]. A face-to-face discussion between the two reviewers took place to reach an agreement on the methodological quality assessment. The PEDro scale has 11 items consisting of risk of bias on randomization, allocation concealment, blinding, dropout rate, intention to treat, and data reporting. Aside from the first item, each of the remaining 10 items is scored 1 mark if a clinical controlled trial meets the criterion, and the final score is obtained by summation. Studies with a PEDro score of 9–10 are considered to be of “excellent” quality, 6–8 of “good” quality, 4–5 of “fair” quality, and below 4 of “poor” quality [43].

### Data synthesis

With reference to Chhatbar et al.’s interpretation of why to use mean change scores, rather than post-intervention outcomes, in meta-analyses [44], we decided to use the mean change score and SD of each interested outcome measure for our meta-analysis. If the mean change score and SD were not available, but the assessment results regarding pre-intervention and post-intervention/follow-up were available, we transformed the pre/post-intervention scores to a mean change score and SD following the recommendation in the Cochrane Handbook for Systematic Reviews of Interventions [45].

Among the included clinical studies, the Fugl-Meyer Assessment - Upper Extremity (FMA-UE) score, which consists of continuous data, was the most common primary outcome measure for upper extremity function. However, there were two studies in which the authors employed the Manual Function Test [46] and the Jebsen Hand Function Test [47] as the primary outcomes, rather than the FMA-UE. To combine the two outcomes in our meta-analysis, we adopted the standardized mean difference (SMD) with 95% confidence intervals (CI) as the pooled effect size. Heterogeneity across the included studies was confirmed by means of checking the Higgins’ *I*^*2*^ statistic. A fixed-effects model for data pooling was used if the *I*^*2*^ statistic was below 50%, which meant that there was acceptable heterogeneity across the included studies. In contrast, the random-effects model was used if the *I*^*2*^ statistic was above 50%. Random-effects models for sub-group analyses among the devices combined with BCIs (e.g., FES, robots, visual feedback) and the effects of differential BCI tasks (e.g., MI-, movement attempt-, and action observation-based BCIs) were conducted. We also conducted a sensitivity analysis by only including studies with good or above methodological quality, to test the robustness of the estimation of effect sizes. Publication basis was checked for through a meta-analysis or subgroup analysis including five or more studies, using Egger’s linear regression test to quantify the asymmetry of the funnel plot. Univariate meta-regression analysis was performed when using the total number of training sessions and the cumulative training time (hours) to identify any association between training dosage and effect size. The level of significance was set at *p* < 0.05 for all statistical analyses performed. Procedures related to data pooling were carried out in Review Manager 5.3 [48], and Comprehensive Meta-Analysis 3.0 software (Englewood, NJ, USA) was used for publication bias and meta-regression.

We also summarized the adverse events of BCI training and the neural mechanism of BCI training reported in the included studies. To create a systematic qualitative description, we considered both the consistency of results across the included studies and the heterogeneity in methodological quality and sample size. However, the single-group studies were qualitatively described only.

## Results

### Literature search and study characteristics

**Fig. 1:**
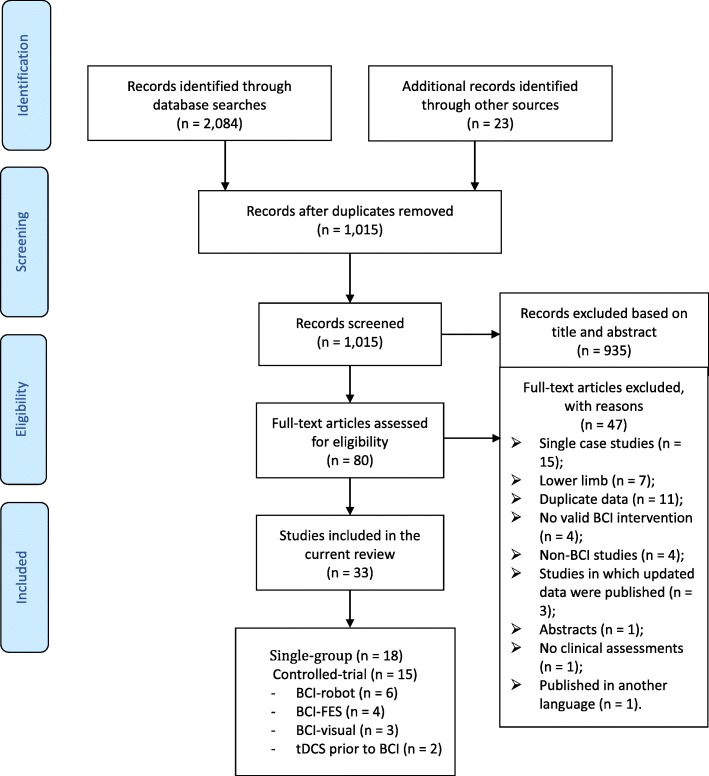
Flow chart of study selection

A total of 1015 records were screened, of which the full texts of 80 were assessed for eligibility. Finally, 33 studies were included in the current systematic review [6, 7, 15–17, 26, 35, 37, 46, 47, 49–71], of which 18 studies were of single-group design [26, 37, 56–71], and 15 studies were of controlled-trial design [6, 7, 15–17, 35, 46, 47, 49–55]. In the current review, only studies with a controlled-trial design were included in our meta-analysis, and those with single-group designs were only included in our qualitative description. A flowchart depicting the study selection is presented in Fig. 1. The characteristics of the included single-group and controlled studies are presented in Tables 1 and 2, respectively. All of the included controlled studies were randomized controlled trials except two [49, 55]. Thirteen out of the 15 controlled studies focused on the effects of BCIs in stroke [6, 7, 15–17, 35, 46, 47, 49–53], while the remaining two studies explored the effects of tDCS in facilitating the effects of BCIs on the improvement of motor recovery in the hemiparetic upper extremity [54, 55]. The methodological quality of the included controlled studies is presented in Table 3. Ramos-Murguialday and colleagues published two papers, in 2013 [15] and 2019 [53], respectively, based on a single experiment. The first paper focused on the immediate effects post-intervention, while the latter focused on the long-term effects of BCIs. We used the data from the earlier study in our meta-analysis of immediate effects, while the latter study was used in our meta-analysis of long-term effects. The EEG signal was commonly used to drive external devices, except for one study using the NIRS signal [35] and another one using the MEG [56]. The majority of the included studies used the signals of electrodes on the ipsilesional hemispheres – in particular, the sensorimotor cortex [6, 15, 16, 47, 55] – while Mihara et al. used the signal from PMC [35] and both Kim et al. and Jang et al. used the EEG signal from the prefrontal cortex [6, 51]. Six studies investigated the neural mechanism behind clinical effects via fMRI, EEG, TMS, or fNIRS [6, 15–17, 35, 49].

**Table 1:** Characteristics of the single-group studies

Study, year	n	Age (years)^a^	Time since stroke^a^	Brain signal for BCI	BCI intervention	Dosage of BCI	Outcome measures	Main results
Buch et al. (2008) [56]	8	58.2 ± 7.0	25.2 ± 11.6 (mo)	MEG, mu	BCI-orthosis	1–2 h/d, 3–5 d/wk., 3–8 wk	MRC	Increased mu rhythm modulation, but no clinical change in MRC.
Prasad et al. (2010) [26]	5	58.6 ± 8.98	28 ± 15.4 (mo)	EEG, mu, beta	BCI-visual feedback	30 min/d, 2 d/wk., 6 wk	ARAT, MRC, 9-HPT	Positive improvement in at least one outcome in all subjects.
Tung et al. (2013) [57]	6	Unknown	Unknown	EEG	BCI-robot	1 h/d, 5 d/wk., 2 wk	FMA-UE	Significant improvement in FMA-UE after BCI.
Ono et al. (2014) [37]	12	57.6 ± 11.8	30.8 ± 41.3 (mo)	EEG, mu, beta	BCI-visual feedback/somatosensory feedback	1 h/d, 12–20 d	SIAS, EMG	BCI training with somatosensory feedback was more effective than that with visual feedback.
Morone et al. (2015) [58]	8	60 ± 10.9	24.4 ± 21.2 (wk)	EEG, beta	Con-rehab + BCI-visual feedback	30 min/d, 3 d/wk., 4 wk	FMA-UE, NIHSS, BI	Positive improvement in all subjects and half of them had improvements higher than the MCID.
Kawakami et al. (2016) [59]	29	50.6 ± 10.9	48 ± 41.4 (mo)	EEG, mu	40 min standard training + BCI-orthosis	45 min/d, 5 d/wk., 2 wk	FMA-UE, MAL, MAS	Significant improvement in FMA-UE and MAL scores after BCI training.
Kotov et al. (2016) [60]	5	47.0 ± 7.7	2 mo-4 yr	EEG	BCI-exoskeleton	8–10 d	NIHSS, MAS, BI, mRS	All patients showed decreases in neurological deficit after BCI training.
Bundy et al. (2017) [61]	10	58.6 ± 10.3	73.6 ± 104.2 (mo)	EEG, mu, beta	BCI-exoskeleton	10–120 min/d, 5 d/wk., 12 wk	ARAT, MAS,	Significant improvement in ARAT after BCI training.
Ibáñez et al. (2017) [62]	4	54.3 ± 11.8	4 ± 0.8 (yr)	EEG, 7–30 Hz, Bereitschafts potential	BCI-FES	10 days in one month	FMA-UE	Improved scores in FMA-UE after BCI training.
Sullivan et al. (2017) [63]	6	57.5 ± 7.9	51.5 ± 41.9 (mo)	EEG, MRCP	BCI-exoskeleton	12 d in 5 wk	FMA-UE	Significant improvement in FMA-UE after BCI training.
Nishimoto et al. (2018) [64]	26	50.2 ± 11.1	47.4 ± 43.9 (mo)	EEG, mu	BCI-exoskeleton + FES	40 min/d, 10 d	FMA-UE, MAL	Significant improvement in FMA-UE and MAL after BCI training.
Chowdhury et al. (2018) [65]	4	44.75 ± 15.69	7 ± 1.15 (mo)	EEG, mu, low beta	BCI-exoskeleton	2–3 d/wk., 6 wk	ARAT, GS	The group mean changes from baseline in GS and ARAT were + 6.38 kg and + 5.66, respectively.
Norman et al. (2018) [66]	8	59.5 ± 11.8	At least 6 (mo)	EEG, mu, beta	BCI- visual feedback	3 d/wk., 4 wk	BBT	Hand function, measured by BBT improved by 7.3 ± 7.5 versus 3.5 ± 3.1 in those with and without SMR control.
Remsik et al. (2018) [67]	21	61.6 ± 15	1127 ± 1327 (d)	EEG	BCI- visual feedback, FES	2 h/d, 15 d	ARAT, 9-HPT, SIS	Significant improvement in ARAT after BCI training.
Tabernig et al. (2018) [68]	8	61.2 ± 19.0	36.8 ± 24.2 (mo)	EEG, beta	BCI-FES	1 h/d, 4 d/wk., 5 wk	Modified FMA-UE	Significant improvement in modified FMA-UE after BCI training.
Carino-Escobar et al. (2019) [69]	9	58.1 ± 12.1	158 ± 74 (d)	EEG, mu, beta	BCI-orthosis	3 d/wk., 4 wk	FMA-UE	Six out of nine subjects had higher scores in FMA-UE after BCI training.
Foong et al. (2019) [70]	11	55.2 ± 11.0	333.7 ± 179.6 (d)	EEG	Standard arm therapy + BCI-visual feedback	1 h/d, 2 d/wk., 6 wk	FMA-UE, ARAT	Significant improvement in FMA-UE after BCI training.
Rathee et al. (2019) [71]	4	62.5 ± 5.7	23 ± 4.2 (mo)	EEG, EMG	BCI-exoskeleton	6 wk	ARAT, GS	Significant improvement in ARAT and GS after BCI training.

^a^Data is reported as means (SD)

*mo* month(s), *yr* year(s), *wk*. weak(s), *h* hour(s), *d* day(s), *BCI* Brain-computer interface, *MEG* Magnetoencephalography, *MRC* Medical Research Council scale, *SIAS* Stroke Impairment Assessment Set, *EEG* Electroencephalography, *ARAT* Action Research Arm Test, *9-HPT* Nine-Hole Peg Test, *NIHSS* National Institute of Health Stroke Scale, *BI* Barthel Index, *EMG* Electromyography, *SMR* Sensorimotor rhythm, *FES* Functional electrical stimulation, *SIS* Stroke Impact Scale, *con-rehab* conventional rehabilitation, *FMA-UE* Fugle-Meyer assessment-upper extremity, *MCID* Minimal clinically important difference, *MAL* Motor activity log, *GS* Grip strength, *MAS* Modified Ashworth scale, *mRS* modified Rankin scale, *MRCP* Movement-related cortical potentials, *BBT* Box and Block Test

**Table 2:** Characteristics of the controlled studies

Study, year	Design	n (E/C)	Age (years)^a^	Time since stroke^a^	Brain signal for BCI	Experimental group	Control group	Dosage of BCI	Outcome measures
Mihara et al. (2013) [35]	RCT	10/10	E: 56.1 ± 7.9 C: 60.1 ± 8.5	E: 146.6 ± 36.2 (d) C: 123.4 ± 38.3 (d)	NIRS, oxyHB	Con-rehab + BCI-visual feedback (MI task)	Con-rehab + sham BCI	20 min/d, 3 d/wk., 2 wk., 6 d	FMA-UE, ARAT, MAL, fNIRS
Ramos-Murguialday et al. (2013) [15]	RCT	16/16	E: 49.3 ± 12.5 C: 50.3 ± 12.2	E: 66 ± 45 (mo) C: 71 ± 72 (mo)	EEG, beta	1 h PT rehab + BCI-orthosis (MA task)	1 h PT rehab + 1 h sham BCI	40 min/d, 5 d/wk., 4 wk., 20 d	FMA-UE, GAS, MAL, MAS, task-fMRI
Varkuti et al. (2013) [49]	NRCT	6/3	E: 40.94 ± 14.5 C: 50.67 ± 6.66	E: 11.67 ± 13.51 (mo) C: 6.8 ± 6.5 (mo)	EEG	BCI-Manus robot (MI task)	Manus robot	1 h/d, 3 d/wk., 4 wk., 12 d	FMA-UE, RS-fMRI
Ang et al. (2014) [50]	RCT	6/8	E: 54.1 ± 8.9 C: 51.1 ± 6.3	E: 258.7 ± 64.0 (d) C: 398.2 ± 150.9 (d)	EEG,	0.5 h mobilization + BCI-robot (MI task)	0.5 h mobilization + robot	1.5 h/d, 3 d/wk., 6 wk., 18 d	FMA-UE,
Li et al. (2014) [6]	RCT	7/7	E: 66.3 ± 4.9 C: 67.1 ± 6.0	E: 2.2 ± 1.8 (mo) C: 2.8 ± 2.0 (mo)	EEG, mu, beta	Con-rehab + BCI-FES (MI task)	Con-rehab + FES	1–1.5 h/d, 3 d/wk., 24 d,	FMA-UE, ARAT, EEG
Rayegani et al. (2014) [47]	RCT	10/10	E: 51 ± 7.3 C: 54 ± 8.2	E: 8.5 ± 6 (mo) C: 8 ± 8.8 (mo)	EEG, beta	1 h con-rehab + BCI-visual feedback (MI task)	Con-rehab	30 min/d, 5 d/wk., 2 wk., 10 d	JHFT
Ang et al. (2015) [7]	RCT	11/14	E: 48.5 ± 13.5 C: 53.6 ± 9.5	E: 383.0 ± 290.8 (d) C: 234.7 ± 183.8 (d)	EEG, FBCSP	BCI-Manus robot (MI task)	Manus robot	1.5 h/d, 3 d/wk., 4 wk., 12 d	FMA-UE
Pichiorri et al. (2015) [17]	RCT	14/14	E: 64.1 ± 8.4 C: 59.6 ± 12.7	E: 2.7 ± 1.7 (mo) C: 2.5 ± 1.2 (mo)	EEG, 0–60 Hz	3 h con-rehab + BCI-visual feedback (MI task)	3 h con-rehab + MI	30 min/d, 3 d/wk., 4 wk. 12 d	FMA-UE, MRC, MAS, NIHSS, EEG
Jang et al. (2016) [46]	RCT	10/10	E: 61.10 ± 13.77 C: 61.70 ± 12.09	E: 4.40 ± 0.97 (mo) C: 4.10 ± 0.74 (mo)	EEG, (SMR + mid-beta) / theta	30 min con-rehab + BCI-FES (AO task)	30 min con-rehab + FES	20 min/d, 5 d/wk., 6 wk., 30 d	VD, HD, VAS, MAS, MFT
Kim et al. (2016) [51]	RCT	15/15	E: 59.09 ± 8.07 C: 59.93 ± 9.79	E: 8.27 ± 1.98 (mo) C: 7.80 ± 1.78 (mo)	EEG, (SMR + mid-beta) / theta	30 min con-rehab + AO-BCI-FES (AO task)	30 min con-rehab	30 min/d, 5 d/wk., 4 wk., 20 d	FMA-UE, MAL, MBI, ROM
Frolov et al. (2017) [52]	RCT	55/19	E: 55.0 ± 12.9 C: 58.5 ± 10.9	E: 8.9 ± 6.4 (mo) C: 8.8 ± 8.4 (mo)	EEG, 5–30 Hz	Con-rehab + BCI-arm exoskeleton (MI task)	Con-rehab + sham BCI	30 min/d, 3 d/wk., 12 d	FMA-UE, ARAT,
Biasiucci et al. (2018) [16]	RCT	14/13	E: 56.4 ± 9.9 C: 59.0 ± 12.4	B: 39.8 ± 45.9 (mo) C: 33.5 ± 30.5 (mo)	EEG, mu, beta	BCI-FES (MA task)	Sham BCI	1 h/d, 2 d/wk., 5 wk., 10 d	FMA-UE, MRC, MAS, ESS, EEG,
Ramos-Murguialday et al. (2019) [53]	RCT	16/12	E: 49.3 ± 12.5 C: 50.3 ± 12.2	E: 66 ± 45 (mo) C: 71 ± 72 (mo)	EEG, beta	1 h PT rehab + 1 h BCI-orthosis (MA task)	1 h PT rehab + 1 h sham BCI	1 h/d, 5 d/wk., 4 wk., 20 d	FMA-UE, GAS, MAL, MAS, task-fMRI
Ang et al. (2015) [54]	RCT	10/9	E: 52.1 ± 11.7 C: 56.3 ± 9.5	E: 1052 ± 722 (d) C: 1021 ± 465 (d)	EEG	20 min tDCS + BCI- robot (MI task)	20 min sham tDCS + BCI- robot	1 h/d, 5 d/wk., 2 wk	FMA-UE
Kasashima-Shindo et al. (2015) [55]	NRCT	11/7	E: 53.5 ± 12.4 C: 48 ± 9.7	E: 46.2 ± 20.2 (mo) C: 56.4 ± 36.4 (mo)	EEG, mu	10 min tDCS + BCI-orthosis (MI task)	BCI-orthosis	45 min/d, 5 d/wk., 2 wk	FMA-UE, MAS

^a^Data is reported as means (SD)

*RCT* Randomized control trial, *NRCT* Non-randomized control trial, *E* Experimental group, *C* Control group, *BCI* Brain-computer interface, *NIRS* Near-infrared spectroscopy, *oxyHB* oxygenated hemoglobin, *con* conventional, *MI* Motor imagery, *AO* Action observation, *MA* Movement attempt, *rehab* rehabilitation, *min* minute(s), *h* hour(s), *d* day(s), *wk.* week(s), *mo* month(s), *yr* year(s), *FMA* Fugl-Meyer assessment, *UE* Upper extremity, *LE* lower extremity, *ARAT* Action research arm test, *MAL* Motor activity log, *MAS* Modified Ashworth scale, *fNIRS* functional near-infrared spectroscopy, *EEG*Electroencephalography, *SMR* Sensorimotor rhythm, *PT* Physical therapy, *GAS* Goal attainment scale, *fMRI* functional magnetic resonance imaging, *RS* Resting state, *FES* functional electrical stimulation, *JHFT* Jebsen Hand Function Test, *MRC* Medical Research Council scale, *NIHSS* National Institute of Health Stroke Scale, *VD* Vertical distance, *HD* Horizontal distance, *AO* Action observation, *VAS* Visual analogue, *MFT* The Manual Function Test, *MBI* Modified Barthel Index, *ROM* Range of motion, *ESS* European Stroke Scale, *tDCS* transcranial direct current stimulation, *RMT* Resting motor threshold, *SICI* Short intra-cortical inhibition, *ICF* Intracortical facilitation

**Table 3:** Methodological quality assessment of the controlled studies

Authors	PEDro items												Total
	1	2	3	4	5	6	7	8	9	10	11		
Mihara et al. (2013) [35]	1	1	1	1	1	1	1	1	1	1	1	10	
Ramos-Murguialday et al. (2013) [15]	1	1	1	1	1	1		1		1	1	8	
Varkuti et al. (2013) [49]	1			1				1	1		1	4	
Ang et al. (2014) [50]	1	1		1			1	1		1	1	6	
Li et al. (2014) [6]	1	1	1	1				1	1	1	1	7	
Rayegani et al. (2014) [47]	1	1		1			1			1	1	5	
Ang et al. (2015) [7]	1	1	1	1			1	1		1		6	
Pichiorri et al. (2015) [17]	1	1	1	1				1		1	1	6	
Jang et al. (2016) [46]	1	1		1			1	1		1	1	6	
Kim et al. (2016) [51]	1	1	1	1			1	1		1	1	7	
Frolov et al. (2017) [52]	1	1		1			1			1	1	5	
Biasiucci et al. (2018) [16]	1	1	1	1	1		1	1	1	1	1	9	
Ramos-M et al. (2019) [53]	1	1	1	1	1	1	1			1	1	8	
Ang et al. (2015) [54]	1	1		1	1			1	1	1	1	7	
Kasashima-Shindo et al. (2015) [55]	1			1			1	1	1	1	1	6	

1 = eligibility criteria; 2 = random allocation; 3 = concealed allocation; 4 = baseline comparability; 5 = blind subjects; 6 = blind therapists; 7 = blind assessors; 8 = adequate follow-up; 9 = intention-to-treat analysis; 10 = between-group comparisons; 11 = point estimates and variability

### Adverse events

Eight of the included studies (*n* = 33) announced that no serious adverse events were found while applying BCIs in patients after stroke [6, 7, 15, 16, 35, 50, 52, 64]. However, it is noteworthy that a few subjects reported mild discomfort after receiving BCI training, such as transient nausea [7], fatigue [7, 52], headaches [52], increased blood pressure [52], and allergies to electrode slices [6]. In Ang et al.’s study, the authors had already excluded patients with epilepsy, but one case dropped out due to a mild transient seizure occurring several hours after the intervention [6].

### Single-group studies

Eleven of the 18 single-group studies evaluated the effects of BCIs on the improvement of the motor recovery of the upper extremities [37, 57, 59, 61, 63, 64, 66–68, 70, 71], and all of them showed significant improvements in motor function, as measured by the FMA-UE, the Action Research Arm Test, and the Box and Block Test. In particular, the average duration since stroke onset indicated that the subjects were at chronic stages, and so improvements in motor function were less likely to be caused by spontaneous recovery. In those single-group studies in which statistical analysis had not been performed, most likely due to the small sample size, descriptive statistics indicated that the majority of subjects benefited from the BCI treatment [26, 37, 58, 60, 62, 65, 69]. However, Buch et al. [56] found that six out of eight subjects could volitionally control the BCI system via the ERD of the mu rhythm, whereas there was no significant improvement in residual finger extension, as measured by the Medical Research Council Scale, after 13 to 22 training sessions. One reason for this might be that all subjects were unable to move their paretic hand because of severe hemiplegia. Another reason for this might be the way in which the outcome measure, the Medical Research Council Scale, which measures gross hand motor function, was not sensitive to minor recovery in hands caused by the BCI training.

### Controlled-trial studies

#### Immediate effects on upper extremity motor function

**Fig. 2:**
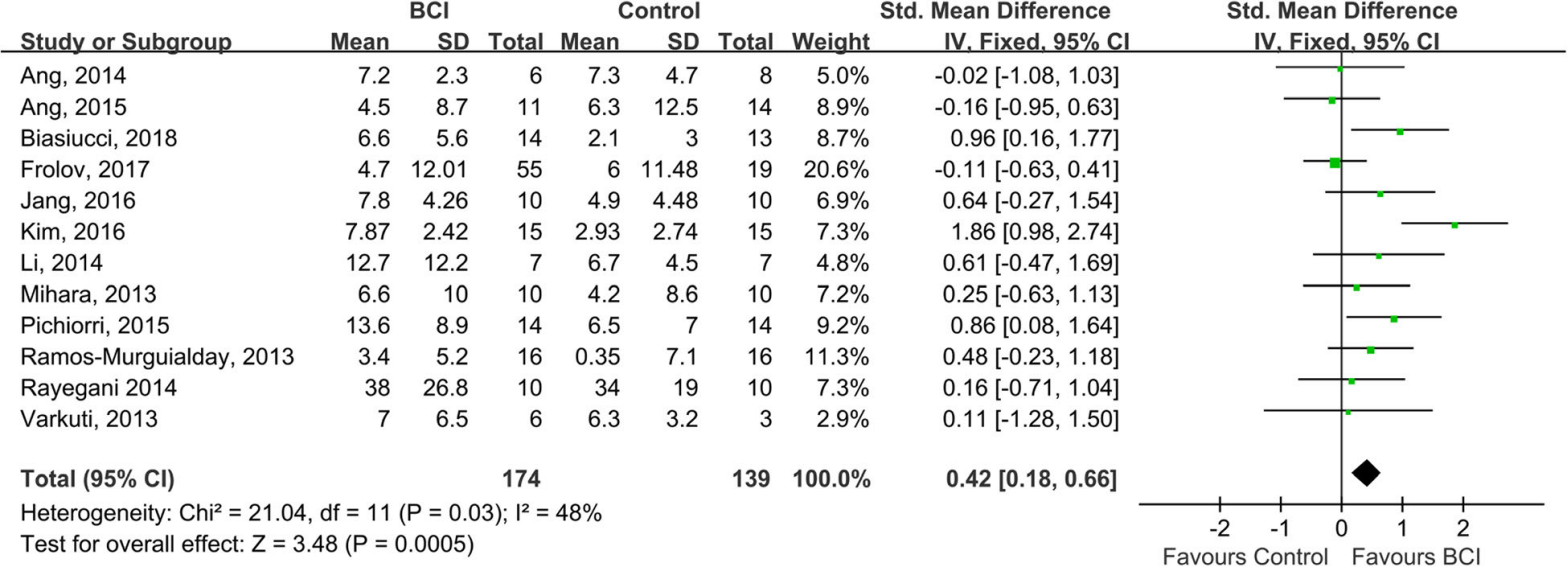
Comparison of the immediate effects of BCI interventions and control interventions on upper extremity motor function. The change in scores and standard deviations (SD) of both BCI and control groups in the 12 included studies were pooled and the overall effect of the BCIs was computed as a standard mean difference (SMD) with 95% confidence interval. The results indicated that BCI training was significantly effective at improving upper extremity function SMD = 0.42; 95% CI = 0.18–0.66; I2 = 48%; *P* < 0.001; fixed-effects model)

**Fig. 3:**
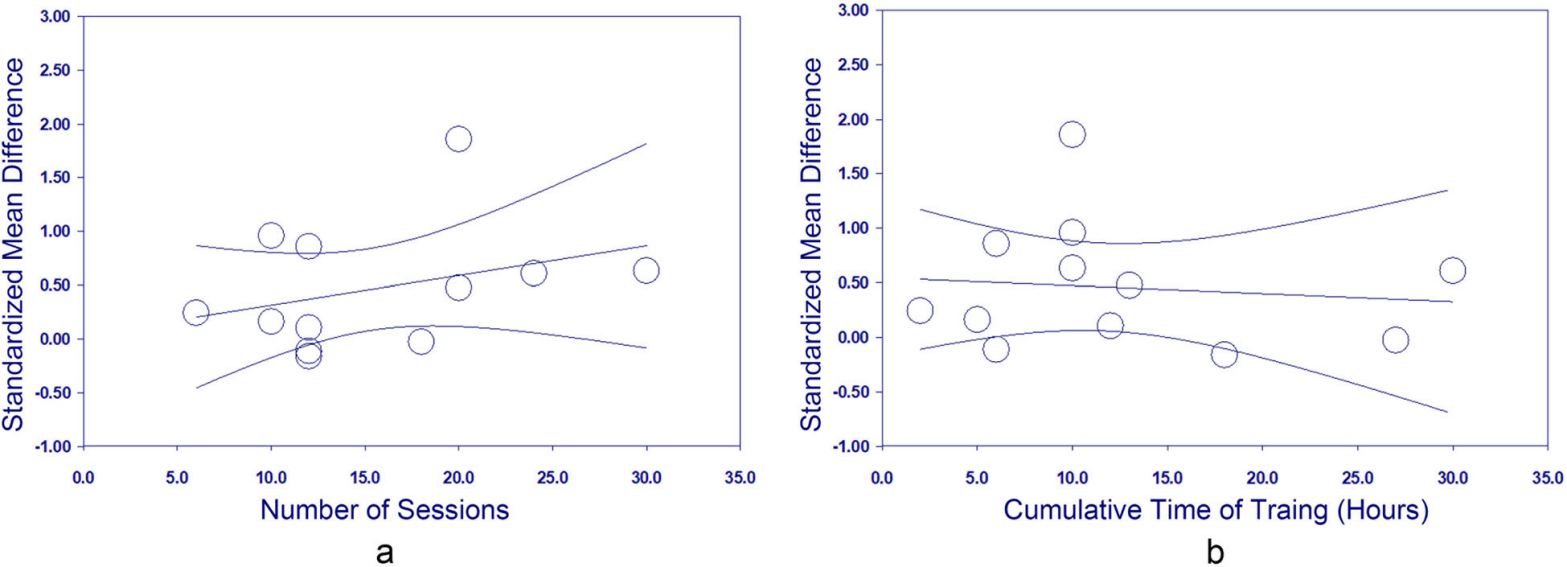
Meta-regression scatterplots show the relationship between the effect size (standardized mean difference) and number of sessions (**a**), and the cumulative training time (**b**). In each subplot, the straight line shows the regression line and the curves around it show the 95% confidence interval

In total, 174 and 139 patients from 12 studies were included in the BCI group and the control group, respectively. The PEDro scores ranged from 4 to 10, with an average score of 6.6 ± 1.7 (Table 3). The pooled results showed that BCIs had a significant effect on the improvement of upper extremity function, compared with control interventions (SMD = 0.42; 95% CI = 0.18–0.66; *I*^*2*^ = 48%; *P* < 0.001; fixed-effects model) (Fig. 2). The funnel plot looked generally symmetrical (see supplementary material, Figure S1) and no evidence of publication bias was noted according to the Egger’s test conducted (β = 1.703; standard error = 1.982; *P* = 0.410). The sensitivity analysis showed that the BCI training had significant effects on upper extremity function when only studies with good or above methodological quality were included (SMD = 0.62; 95% CI = 0.33–0.90; *I*^2^ = 46%; *P* < 0.001; fixed-effects model). The meta-regression showed that neither the total number of sessions (β = 0.028, standard error = 0.024, *P* = 0.252) nor the cumulative time of training (β = − 0.007, standard error = 0.021, *P* = 0.732) were significant predictors of the effect size (Fig. 3).

#### Subgroup analysis of the effects of different BCI tasks

**Fig. 4:**
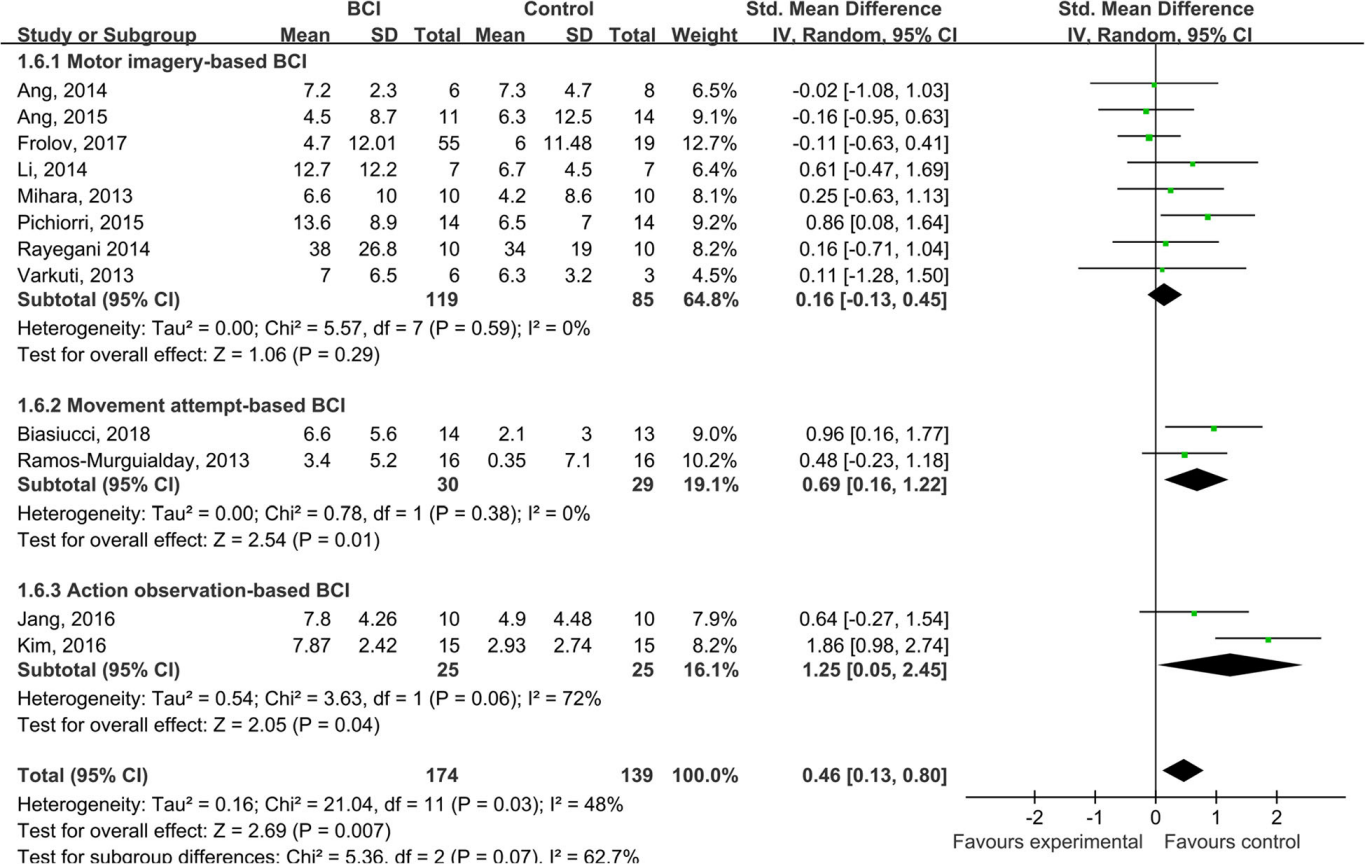
A subgroup analysis for the effects of different BCI mental tasks. The 12 included studies were categorized into motor imagery-based BCIs (eight studies), movement attempt-based BCIs (two studies), and action observation-based BCIs (two studies), depending on the nature of the mental tasks. The results indicted that both movement attempt-based (SMD = 0.69; 95% CI = 0.16–1.22; *I*^2^ = 0%; *P* = 0.010; random-effects model) and action observation-based BCIs (SMD = 1.25; 95% CI = 0.0.05–2.45; *I*^2^ = 72%; *P* = 0.040; random-effects model) tended to show superior clinical effects, compared to MI-based BCIs (SMD = 0.16; 95% CI = − 0.13 – 0.45; *I*^2^ = 0%; *P* = 0.290; random-effects model) in regard to improving upper extremity function. However, the difference among subgroups was not significant (*P* = 0.070)

With regard to the driving tasks of BCIs, MI-based BCIs were the most popular and the ERD in the mu and/or beta frequency bands was used to drive the BCI feedback devices [6, 7, 17, 35, 47, 49, 50, 52]. We also found movement attempt-based BCIs developed in two studies [62, 63]. In studies conducted by Kim et al. and Jang et al., the feedback devices were driven by a concentration index that was calculated based on the power of low beta (12–15 Hz), mid-beta (16–20 Hz), and theta bands when subjects were observing movements [46, 51]. A subgroup analysis indicted that both movement attempt-based (SMD = 0.69; 95% CI = 0.16–1.22; *I*^2^ = 0%; *P* = 0.010; random-effects model) and action observation-based (SMD = 1.25; 95% CI = 0.0.05–2.45; I2 = 72%; *P* = 0.040; random-effects model) BCIs tended to show superior clinical effects, compared with MI-based BCIs (SMD = 0.16; 95% CI = − 0.13 – 0.45; I2 = 0%; *P* = 0.290; random-effects model), in regard to the improvement of upper extremity function (Fig. 4). However, the difference among subgroups was not significant (*P* = 0.070). The funnel plot of the subgroup meta-analysis for the effects of MI-based BCIs looked symmetrical (Figure S2) and no evidence of publication bias was found based on the Egger’s test conducted (β = 1.153; standard error = 1.327; *P* = 0.418).

#### Subgroup analysis of the effects of different devices combined with BCIs

**Fig. 5:**
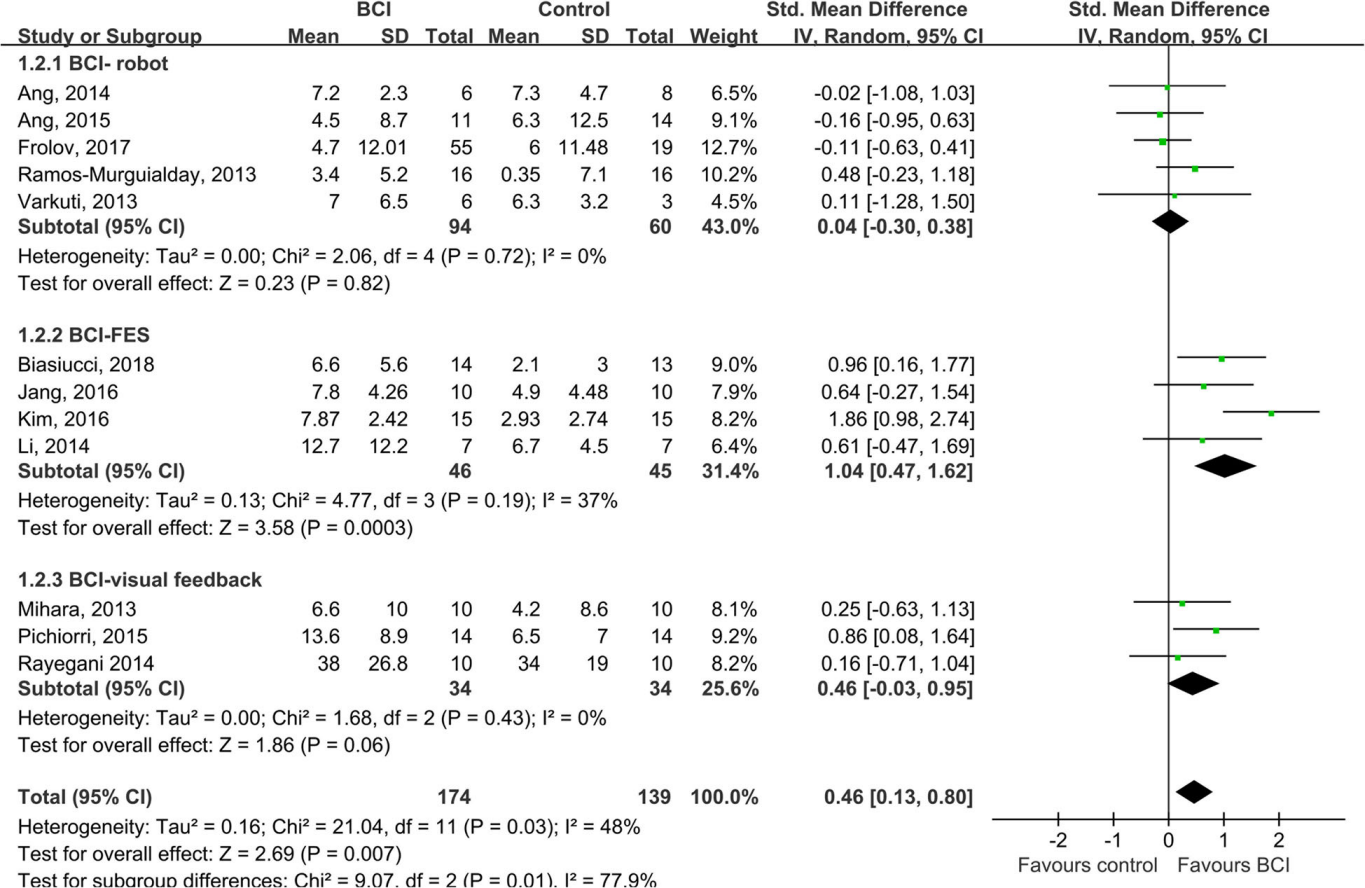
A subgroup analysis of the effects of different devices combined with BCIs. The results indicated that only BCIs triggering the stimulation of FES had a significantly large effect size on motor function recovery, compared with control interventions (SMD = 1.04; 95% CI = 0.47–1.62; I^2^ = 37%; P < 0.001; random-effects model), while both BCIs combined with robots (SMD = 0.04; 95% CI = − 0.30 – 0.38; I^2^ = 0%; *P* = 0.820; random-effects model) and with visual feedback (SMD = 0.46; 95% CI = − 0.03 – 0.95; I^2^ = 0%; *P* = 0.060; random-effects model) had no significant differential clinical effects with control interventions

With regard to BCI feedback devices, upper extremity robot and arm orthosis were most commonly used [7, 15, 49, 50, 52], followed by FES [6, 16, 46, 51], and visual feedback [17, 35, 47]. A subgroup analysis indicated that only FES triggered by BCIs had a significant large effect on motor function recovery, compared with control interventions (SMD = 1.04; 95% CI = 0.47–1.62; *I*^*2*^ = 37%; *P* < 0.001; random-effects model), while neither BCIs combined with robots (SMD = 0.04; 95% CI = − 0.30 – 0.38; *I*^*2*^ = 0%; *P* = 0.820; random-effects model) or visual feedback (SMD = 0.46; 95% CI = − 0.03 – 0.95; *I*^*2*^ = 0%; *P* = 0.060; random-effects model) had significant differential clinical effects with control interventions (Fig. 5). The difference among subgroups was significant (*P* = 0.010). The funnel plot of the subgroup meta-analysis of the effects of BCIs combined with robots looked symmetrical (Figure S3) and no evidence of publication bias was found based on the Egger’s test conducted (β = 0.344; standard error = 1.318; *P* = 0.811). The funnel plots of the subgroup meta-analyses of FES and visual feedback are presented in Figure S4 and Figure S5, respectively.

#### Long-term effects on upper extremity motor function

**Fig. 6:**
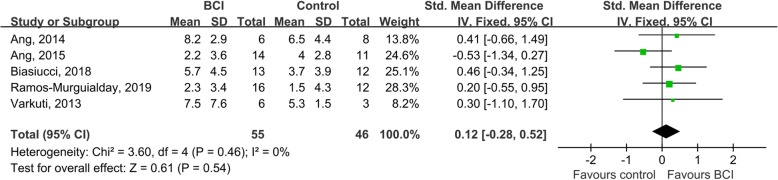
A comparison of the long-term effects of BCI interventions and control interventions on upper extremity motor function. The inputted data consisted of the change in scores between baseline and follow-up. Five studies followed up with patients from between 6 weeks to six-to-12 months. Ang et al. followed up with subjects twice after the intervention, after 6 weeks and after 18 weeks [48]. Our meta-analysis indicated that BCIs did not show any significant differential effects compared with control interventions when we used the follow-up data from Ang et al.’s study at 6 weeks [48] (SMD = 0.12; 95% CI = − 0.28 – 0.52; *I*^2^ = 0%; *P* = 0.540; fixed-effects model)

Five studies consisting of one of excellent quality [16], three of good quality [7, 50, 53], and one of fair quality [49], evaluated the long-term effects of BCIs on upper extremity motor function. However, the follow-up times were inconsistent, ranging from 6 weeks [50], to 8 weeks [7, 49], to six-to-12 months [16, 53]. Ang et al. followed up subjects twice after the intervention, after 6 weeks and after 18 weeks [50]. Four studies utilized robots as the BCI feedback device [7, 49, 50, 53], while Biasiucci et al. combined BCIs with FES [16]. The meta-analysis indicated that BCIs did not show any significant differential effects compared with control interventions, regardless of whether we used the data from the follow-up at 6 weeks (SMD = 0.12; 95% CI = − 0.28 – 0.52; *I*^2^ = 0%; *P* = 0.540; fixed-effects model) (Fig. 6) or 18 weeks (SMD = 0.11; 95% CI = − 0.29 – 0.51; *I*^2^ = 0%; *P* = 0.590; fixed-effects model) in Ang et al.’s study [50]. The funnel plot of the former looked symmetrical (Figure S6) and no evidence of publication bias was found based on the Egger’s test conducted (β = 1.210; standard error = 2.687; *P* = 0.683). A similar result was also identified, in that BCIs combined with robots had comparable effects with those of the interventions in the control groups (SMD = 0.01; 95% CI = − 0.45 – 0.47; *I*^2^ = 0%; *P* = 0.960; fixed-effects model).

#### Spasticity, strength, activities of daily living, and shoulder subluxation

The spasticity assessment for upper extremities was identified in four studies, one of excellent methodological quality [16] and three of good methodological quality [15, 17, 46]. Nevertheless, none of the studies showed any significant differences in regard to the spasticity between groups after the interventions. Two studies found that the BCI groups recovered more in terms of muscle strength than the control groups [16, 17]. In addition, a significant effect on the activities of daily living, measured by the Modified Barthel Index, was found in one study [51]. Jang et al. applied a BCI triggering FES in patients with shoulder subluxation after stroke; the results showed that the BCI group made more significant improvements in the distance from the inferior acromial to the central point of the humeral head than the control group, but the improvement in regard to pain intensity was comparable between the groups [46].

#### Potentiating effects of tDCS on BCI training

**Fig. 7:**
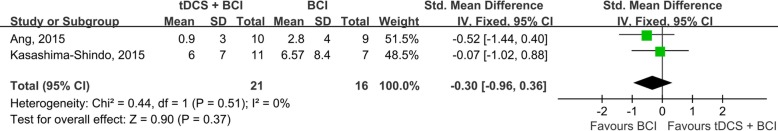
The potentiating effects of tDCS on BCI training in regard to improving upper extremity function. The meta-analysis indicated that the tDCS could not further potentiate the clinical effects of BCIs in regard to improving upper extremity motor function in patients with stroke (SMD = − 0.30; 95% CI = − 0.96 – 0.36; *I*^*2*^ = 0%; *P* = 0.370; fixed-effects model)

Two studies, which included 21 and 16 patients in the experimental and control groups, respectively [54, 55], have explored the potentiating effects of tDCS on BCI training. The meta-analysis, presented in Fig. 7, indicated that tDCS could not further facilitate the clinical effects of BCIs in regard to improving hemiparetic upper extremity motor function in patients with stroke (SMD = − 0.30; 95% CI = − 0.96 – 0.36; *I*^2^ = 0%; *P* = 0.370; fixed-effects model), compared with sham tDCS.

#### Neural mechanism behind the clinical effects

The neural mechanism underlying the clinical effects of BCIs was evaluated in six studies covering cortical activation, excitability of the corticospinal tract, interhemispheric imbalance, and functional connectivity change [6, 15–17, 35, 49]. An fNIRS study showed that the activation of the ipsilesional premotor cortex (PMC) significantly increased after BCI training, while no significant change was observed in the control group [35]. Both Li et al. [6] and Pichiorri et al. [17] identified the way in which the ipsilesional hemisphere, especially the sensorimotor cortex, had more robust desynchronized activity during MI tasks involving affected hands in the BCI group than in the control group [17]. Moreover, BCIs were shown to be effective in rebalancing interhemispheric activities [15].

Functional connectivity change (FCC) was measured in three studies based on fMRI [49] and EEG [16, 17]. The fMRI study was of fair quality, due to non-randomization and a small sample size, but showed that the FCC during resting states was consistently higher in the BCI group than in the control group [49]. However, these statistical analyses were not significant. The two studies based on EEG showed conflicting results, in that Biasiucci et al. found that BCI training could significantly promote higher FCC among the motor network system during resting states [16]. Pichiorri et al., however, found that interhemispheric connectivity density did not differ significantly between or within groups before and after training [17]. However, the study found that there was a significant positive correlation between the increase in ipsilesional connectivity at rest and functional improvement in the BCI group; this relationship could not be found in the control group [17].

## Discussion

The present systematic review and meta-analysis summarizes the safety profiles, clinical effects, and neural mechanism underlying the clinical effects of BCI training in regard to stroke. In this systematic review, we have included both single-group and controlled studies. We have also included updated data published in recent issues and excluded old data to avoid duplicating the inclusion of the same cohorts. The overall methodological quality of the included studies was good. Two of them in particular were rated as being of excellent quality by the PEDro scale [16, 35].

### Adverse events

First, we checked the safety of BCIs for patients after stroke. All studies announced that BCIs were safe, and there were no severe adverse events after receiving BCI treatment. However, some symptoms of discomfort, such as transient nausea [7], fatigue [7, 52], and headaches [52], were reported in a few subjects. Fatigue is one of the most common symptoms post stroke, with a high prevalence ranging from 29 to 77% [72]. One type of fatigue is induced by psychological problems that may be associated with low motivation and post-stroke depression [72]. In many of the included studies, researchers excluded patients with post-stroke depression [7, 15, 16, 50]. Another is exertion fatigue, which may be worsened by intensive physical exercise and mental effort [72]. The BCI training includes mental practices and patients need to concentrate hard on the instructions and feedback to reach a high level of self-regulation [25]. In particular, Frolov et al. found that the majority of patients reported fatigue after 20 to 30 min of training [52]. Therefore, to avoid fatigue during the BCI training, a short rest period after 15 to 20 min of training may be practical.

### Clinical effects

The included single-group studies indicated that patients with stroke can benefit from various kinds of BCI designs in terms of improving the motor recovery of the hemiparetic upper extremity. It is noteworthy that patients with stroke at the chronic stage could also make improvements in their motor recovery, which expands the significance of BCIs in stroke rehabilitation. Our meta-analysis found that the immediate effects on upper extremity function showed a favorable medium effect (SMD = 0.42) induced by BCI training [73], which is lower than that of a previous meta-analysis (SMD = 0.79) [20]. The source of the discrepancy comes from two studies [50, 52]. The first, conducted by Ang et al., completed a randomized controlled trial employing three groups: a BCI-based haptic knob robot group, a haptic knob robot group, and a standard arm treatment group [50]. The results of the FMA-UE indicated that the haptic knob group showed the most significant improvement (7.3 ± 4.9), followed by the BCI-based haptic knob robot group (7.2 ± 2.3) and the standard arm treatment group (4.9 ± 4.1). In the previous meta-analysis, the authors regarded the standard arm treatment group as the control group. Thus, a large effect size was observed. However, in our meta-analysis, we considered the haptic knob robot group as the control group, based on the principle of the worst-case scenario. Thus, the pooled effect size in our meta-analysis was lower than that of the previous one [20]. The second study, conducted by Frolov et al., published updated data in 2017 [52], but the search date of the previous meta-analysis was up to December 2016 [20]. Therefore, in our study, we included the latest published data and excluded the old data [74]. If the two studies are excluded from our current meta-analysis, a high SMD is obtained (SMD = 0.60), which is close to the SMD reported in the previous meta-analysis (SMD = 0.79). Moreover, our sensitivity analysis included only studies with good or above methodological quality, which also indicated a medium effect size (SMD = 0.62). Taken together, these previously mentioned studies prove that the results of our meta-analysis are more robust, indicating a medium effect size in favor of the effects of BCIs on upper extremity function in patients after stroke.

To our surprise, the meta-regression showed that the number of training sessions and the cumulative duration of training were not significant predictors of effect size. The first explanation for this phenomenon might be the heterogenicity of external devices driven by BCIs. As we reported, the devices may be a key factor affecting the clinical effects. Therefore, the dose-effect relationship may be masked in this situation. The second explanation may be the potential existence of a non-linear improvement rate. Most forms of BCI training introduced in the included studies were based on MI. Patients might have difficulty controlling the devices at the beginning, as reflected by the low accuracy rate, which may be due to the higher level of mental effort required at this stage. Our subgroup meta-analysis also indicated that using MI as the mental task for BCIs might not produce the optimal effects, compared with action observation or movement attempts. However, subjects may control devices through MI very easily in the later stages, leading to an increase in accuracy rates. The difference in effort exerted during training may result in a non-linear improvement rate, where there is a greater improvement at the beginning than at the end. Finally, the dosage of BCI training might be suboptimal in some of the included studies, yielding a small effect size, which could affect the results of the meta-regression.

The first subgroup analysis explored the effects of different BCI tasks (e.g., MI-based, movement attempt-based, and action observation-based BCIs) on the improvement in the motor function of the hemiparetic upper extremity. Both the MI-based and movement attempt-based BCIs have previously been widely investigated and differential neural mechanisms have been proposed: MI-related network and Hebbian plasticity theory, respectively [16, 50]. Although studies have indicated that MI seems to share a similar brain network with movement execution [21] and could enhance the cortical excitability of M1 measured by increased MEP [25], many randomized controlled trials with strong methodologies did not support its clinical effects in regard to stroke, as reported in a recent meta-analysis [75]. Among our included studies, two of high quality compared movement-attempted-BCIs with sham BCIs. Our subgroup meta-analysis also showed movement attempt-based BCIs had superior effects, compared to MI-based BCIs. Movement attempt-based BCIs establish a closed sensorimotor loop, which can potentially restore the normal timing order of motor preparation, execution, and peripheral muscle effectors [30]; this form of plasticity may further strengthen corticospinal tract projection [16]. Further studies directly comparing the effects of these two different BCIs are necessary in future.

The second subgroup analysis among the modalities of BCI feedback disclosed an important message. The pooled effect size showed that the BCIs combined with robot training were insignificant [7, 15, 49, 50, 52]. In contrast, BCIs combined with FES had a large effect size, compared with FES alone and other control interventions [6, 16, 46, 51]. The latest Cochrane systematic review reported that robot-assisted arm training had a small effect size (SMD = 0.32) on arm function recovery in patients after stroke, indicated by high-quality evidence [76], while FES seemed to have a greater effect size on upper extremity motor function (SMD = 0.40) [77]. The possible mechanism could be explained by the specific role of FES in somatosensory stimulation. Previous studies in healthy individuals support the notion that neural activation of the primary sensorimotor cortex during motor tasks increases after receiving somatosensory stimulation [78]. Moreover, a study found that the projection of the primary sensory cortex to pyramidal cells of M1 played the role of the “driver” to the M1 [79], which indicates the essential role of somatosensory information in the production of high-quality motor outputs [80, 81]. A BCI system provides a closed-loop pathway from cortical activation to external feedback and then transfers the feedback to the patient’s brain [82]. BCI training can promote activity-dependent plasticity through self-regulated mental activity to produce near-normal brain activity. On the other hand, the combined external device provides sensory input to induce brain plasticity [83]. In summary, these indirect comparisons indicate that BCIs combined with FES may be a better combination than BCIs combined with robots in regard to upper extremity function recovery. Further randomized controlled trials should be conducted in the future to verify this hypothesis.

Only five studies included post-intervention follow-ups at various time points [7, 16, 49, 50, 53]. The pooled effect size showed no significant difference between BCI groups and control groups. The immediate post-intervention effects on upper extremity function were not significantly different between groups in three out of five studies [7, 49, 50]. Thus, it is reasonable to suggest that they had no significant difference at follow-up. Biasiucci et al. found a significantly favorable effect on the BCI group post intervention [30], but the difference in motor function between groups at follow-up was not significant. Therefore, the current review shows that long-term effects of BCI training are not evident. The long-term effects of rehabilitative interventions are important, but most of them were limited in terms of the durability of treatment effects, such as mirror therapy and virtual reality [18, 19].

In the included studies, some secondary outcomes were reported. Consistent results showed that BCI training cannot effectively improve patients’ spasticity. In fact, this result is also consistent with the effects of most conventional treatments [84]. Moreover, muscle strength, activities in daily living, and shoulder subluxation may be effectively improved after BCI training, but the number of studies was quite limited. Moreover, the group difference of mean change scores for the modified Barthel Index was 1.53, which is less than the accepted standard for a clinically meaningful functional improvement [85].

To date, only two studies including 37 patients investigated the potentiating effects of tDCS on MI-based BCI training [54, 55]. Our meta-analysis showed that there were no significant potentiating effects of tDCS on BCI training in terms of improving hemiparetic upper extremity function. On the other hand, both studies found increased ERD during MI, but they reported inconsistent results in regard to improvements in motor performance [54, 55]. Due to the limited number of studies, the effects of adding tDCS before BCI training in motor recovery in stroke are inconclusive and more studies are needed in future.

### Neural mechanism

In addition to spontaneous recovery, the motor recovery of paretic extremities very much depends on the mechanism of neural plasticity at structural and functional levels. Post-stroke rehabilitation training may strengthen connections between neurons in existing neural pathways and lead to the formation of new neural connections [86]. Neural plasticity improvement at the structural level refers to the ability for changes to take place in terms of synapse number and size, receptor density, and the number of neurons in some brain regions [87]. At the functional level, the cortices that are not responsible for given movements may be recruited for movements during the motor recovery stage after a stroke [87]. The latter is known as cortical map reorganization, which is modulated by plenty of training, particularly in the ipsilesional hemisphere, for patients with stroke.

In the current systematic review, seven studies conducted investigations into the neural mechanism of BCIs. Consistent results of two EEG studies showed that, after BCI training, there was a higher power of desynchronization over the ipsilesional central area during MI tasks than pre-intervention, indicating greater activation of the ipsilesional motor system after BCI training [6, 35]. In particular, the PMC was significantly activated, as indicated in an fNIRS study of patients with subcortical stroke [35]. In addition to these randomized controlled trials, there were a large number of studies with pre-post single-group designs involving healthy subjects, which also indicated that BCIs could significantly activate the prefrontal cortex, PMC, and posterior parietal cortex [88, 89]. The PMC is strongly associated with motor planning and the execution of complex and goal-directed actions [90]. In patients after stroke, normal motor planning is disrupted, denoted by the extended processing time in motor planning [91]. Therefore, a limited number of studies pointed out that the potential mechanism was relevant to promoting the motor planning process.

Enhancing the excitability of motor cortex in the ipsilesional hemisphere has been proposed as the neural correlate in terms of the successful motor recovery of the hemiparetic upper extremity [92]. MEP, a quantification for corticospinal excitability, is used to probe the physiology of the motor cortex and the amplitude of MEP is assumed to correlate with motor performance [93]. The reduced amplitude of MEP can be noted after stroke and the absence of MEP over the ipsilesional M1 has been correlated with poor motor recovery and functional outcomes in patients with stroke in the long-term [94]. Mrachacz-Kersting et al. measured MEP to evaluate the effects of BCIs in terms of exciting the corticospinal tract [95]. The amplitude of MEP significantly increased after each session of BCI training, and the effect was sustained even 30 min after the intervention. In contrast, the sham BCI group showed no significant improvements at any level of stimulation intensity [95]. This result is also supported by other researchers [96] and may be another aspect of the neural mechanism of BCI in patients after stroke.

Revision of the interhemispheric imbalance has also been regarded as a target in terms of improving upper extremity function; for example, applying low-frequency rTMS to unaffected M1, Ramos-Murguialday et al. found a shift of activity in the M1 and PMC from the contralesional hemisphere toward the ipsilesional hemisphere during actual finger movements in patients with subcortical lesions, indicating the effects of BCIs in rebalancing interhemispheric activities [15]. However, Young et al. argued that this result could not be generalized to patients with cortical lesions because they found an ipsilesional lateralization during movements of affected hands at baseline toward a bilaterally distributed activity after receiving BCI training in a group of patients in which cortical lesions were involved [97]. A recent longitudinal observational study showed that the interhemispheric inhibition (IHI) was not associated with motor impairment after stroke and, in particular, the IHI was normal in the acute/subacute stage and gradually became abnormal at the chronic stage [98]. Moreover, another meta-analysis summarized the way in which there is no clear evidence for the hyper-excitability of the unaffected hemisphere or imbalanced interhemispheric inhibition [92]. Therefore, caution should be undertaken when interpreting interhemispheric rebalance as one of the mechanisms of motor recovery caused by BCI training.

## Conclusion

BCI training is safe for patients after stroke. The present evidence shows that BCI training has significant immediate effects on the improvement of upper extremity motor function. However, a limited number of studies do not support its long-term effects. Movement attempt-based BCIs seem to be more effective than MI-based BCIs. FES may be a more useful device triggered by BCIs for functional recovery than other kinds of neural feedback. At present, a limited number of studies do not support the role of tDCS in potentiating the effects of BCI training. The neural mechanism of BCIs underlying the clinical effects is very likely to be relevant to the ipsilesional activation in the primary and secondary motor cortices. Even though many studies have been carried out and have shown significant effects of BCI-based rehabilitation on the improvement of upper extremity function, there exists a substantial heterogeneity in terms of the use of mental tasks, feedback devices, and clinical protocols, which deserves further investigation.

## Supplementary information

**Additional file 1: Figure S1.** Funnel plot of the meta-analysis for the immediate effects of BCIs on upper extremity motor function. **Figure S2.** Funnel plot of the subgroup meta-analysis for the effects of motor imagery based BCIs on upper extremity motor function. **Figure S3.** Funnel plot of the subgroup meta-analysis for effects of BCIs combined with robots on upper extremity motor function. **Figure S4.** Funnel plot of the subgroup meta-analysis for the effects of BCIs combined with functional electrical stimulation on upper extremity motor function. **Figure S5.** Funnel plot of the subgroup meta-analysis for the effects of BCIs combined with visual feedback on upper extremity motor function. **Figure S6.** Funnel plot of the meta-analysis for the long-term effects of BCIs on upper extremity motor function.

## Data Availability

The datasets supporting the conclusions of this article are included withinthe article.

## Abbreviations

M1: Primary motor cortex
MI: Motor imagery
BCI: Brain-computer interface
FES: Functional electrical stimulation
EEG: Electroencephalography
MEG: Magnetoencephalography
fNIRS: functional near-infrared spectroscopy
fMRI: functional magnetic resonance imaging
ERD: Event-related desynchronization
ERS: Event-related synchronization
SMA: Supplementary motor area
TMS: Transcranial magnetic stimulation
MEP: Motor evoked potential
tDCS: transcranial direct current stimulation
FMA-UE: Fugl-Meyer Assessment - Upper Extremity
SD: Standard deviation
SMD: Standardized mean difference
CI: Confidence intervals
PMC: Premotor cortex
FCC: Functional connectivity change
IHI: Interhemispheric inhibition

## References

[1] Wolfe CD (2000). The impact of stroke. Br Med Bull.

[2] Cramer SC (2008). Repairing the human brain after stroke: I. mechanisms of spontaneous recovery. Ann Neurol.

[3] Jeon BJ, Kim WH, Park EY (2015). Effect of task-oriented training for people with stroke: a meta-analysis focused on repetitive or circuit training. Top Stroke Rehabil.

[4] Kwakkel G, Veerbeek JM, van Wegen EE, Wolf SL (2015). Constraint-induced movement therapy after stroke. Lancet Neurol.

[5] Sellers EW, Donchin E (2006). A P300-based brain–computer interface: initial tests by ALS patients. Clin Neurophysiol.

[6] Li M, Liu Y, Wu Y, Liu S, Jia J, Zhang L (2014). Neurophysiological substrates of stroke patients with motor imagery-based brain-computer Interface training. Int J Neurosci.

[7] Ang KK, Chua KS, Phua KS, Wang C, Chin ZY, Kuah CW, Low W, Guan C (2015). A randomized controlled trial of EEG-based motor imagery brain-computer interface robotic rehabilitation for stroke. Clin EEG Neurosci.

[8] Miller KJ, Schalk G, Fetz EE, den Nijs M, Ojemann JG, Rao RP (2010). Cortical activity during motor execution, motor imagery, and imagery-based online feedback. Proc Natl Acad Sci U S A.

[9] Birbaumer N, Cohen LG (2007). Brain-computer interfaces: communication and restoration of movement in paralysis. J Physiol.

[10] Wolpaw JR, Birbaumer N, McFarland DJ, Pfurtscheller G, Vaughan TM (2002). Brain-computer interfaces for communication and control. Clin Neurophysiol.

[11] Pfurtscheller G, Lopes da Silva FH (1999). Event-related EEG/MEG synchronization and desynchronization: basic principles. Clin Neurophysiol.

[12] Zhang JJQ, Fong KNK, Welage N, Liu KPY. The activation of the mirror neuron system during action observation and action execution with mirror visual feedback in stroke: a systematic review. Neural Plast. 2018. 10.1155/2018/2321045. 10.1155/2018/2321045 PMC5941778 29853839

[13] Ma T, Li H, Deng L, Yang H, Lv X, Li P, Li F, Zhang R, Liu T, Yao D, et al. The hybrid BCI system for movement control by combining motor imagery and moving onset visual evoked potential. J Neural Eng. 2017;14. 10.1088/1741-2522/aa5d5f. 10.1088/1741-2552/aa5d5f 28145274

[14] Daly JJ, Cheng R, Rogers J, Litinas K, Hrovat K, Dohring M (2009). Feasibility of a new application of noninvasive brain computer interface (BCI): a case study of training for recovery of volitional motor control after stroke. J Neurol Phys Ther.

[15] Ramos-Murguialday A, Broetz D, Rea M, Laer L, Yilmaz O, Brasil FL, Liberati G, Curado MR, Garcia-Cossio E, Vyziotis A (2013). Brain-machine interface in chronic stroke rehabilitation: a controlled study. Ann Neurol.

[16] Biasiucci A, Leeb R, Iturrate I, Perdikis S, Al-Khodairy A, Corbet T, Schnider A, Schmidlin T, Zhang H, Bassolino M (2018). Brain-actuated functional electrical stimulation elicits lasting arm motor recovery after stroke. Nat Commun.

[17] Pichiorri F, Morone G, Petti M, Toppi J, Pisotta I, Molinari M, Paolucci S, Inghilleri M, Astolfi L, Cincotti F (2015). Brain-computer interface boosts motor imagery practice during stroke recovery. Ann Neurol.

[18] Laver KE, Lange B, George S, Deutsch JE, Saposnik G, Crotty M (2017). Virtual reality for stroke rehabilitation. Cochrane Database Syst Rev.

[19] Thieme H, Mehrholz J, Pohl M, Behrens J, Dohle C (2018). Mirror therapy for improving motor function after stroke. Cochrane Database Syst Rev.

[20] Cervera MA, Soekadar SR, Ushiba J, Millan JDR, Liu M, Birbaumer N, Garipelli (2018). Brain-computer interfaces for post-stroke motor rehabilitation: a meta-analysis. Ann of Clin Transl Neurol.

[21] Sharma N, Pomeroy VM, Baron JC (2006). Motor imagery: a backdoor to the motor system after stroke?. Stroke..

[22] Hétu S, Grégoire M, Saimpont A, Coll MP, Eugène F, Michon PE, Jackson PL (2013). The neural network of motor imagery: an ALE meta-analysis. Neurosci Biobehav Rev.

[23] Pfurtscheller G, Brunner C, Schlögl A, Lopes da Silva FH (2006). Mu rhythm (de) synchronization and EEG single-trial classification of different motor imagery tasks. Neuroimage..

[24] Jeon Y, Nam CS, Kim YJ, Whang MC (2011). Event-related (de) synchronization (ERD/ERS) during motor imagery tasks: implications for brain–computer interfaces. Int J Ind Ergonom.

[25] Ruffino C, Papaxanthis C, Lebon F (2017). Neural plasticity during motor learning with motor imagery practice: review and perspectives. Neuroscience..

[26] Prasad G, Herman P, Coyle D, McDonough S, Crosbie J (2010). Applying a brain-computer interface to support motor imagery practice in people with stroke for upper limb recovery: a feasibility study. J Neuroeng Rehabil..

[27] Shoham S, Halgren E, Maynard EM, Normann RA (2001). Motor-cortical activity in tetraplegics. Nature..

[28] Bi G, Poo M (2001). Synaptic modification by correlated activity: Hebb’s postulate revisited. Annu Rev Neurosci.

[29] Lisman J. Glutamatergic synapses are structurally and biochemically complex because of multiple plasticity processes: long-term potentiation, long-term depression, short-term potentiation and scaling. Philos Trans R Soc Lond Ser B Biol Sci. 2017;372:20160260. 10.1098/rstb.2016.0260. 10.1098/rstb.2016.0260 PMC5247596 28093558

[30] Muralidharan A, Chae J, Taylor DM (2011). Taylor, Extracting attempted hand movements from EEGs in people with complete hand paralysis following stroke. Front Neurosci.

[31] Blokland Y, Vlek R, Karaman B, Özin F, Thijssen D, Eijsvogels T, Colier W, Floor-Westerdijk M, Bruhn J, Farquhar J (2012). Detection of event-related desynchronization during attempted and imagined movements in tetraplegics for brain switch control. Conf Proc IEEE Eng Med Biol Soc..

[32] Popović DB (2014). Advances in functional electrical stimulation (FES). J Electromyogr Kinesiol.

[33] Hu XL, Tong RK, Ho NS, Xue JJ, Rong W, Li LS (2015). Wrist rehabilitation assisted by an electromyography-driven neuromuscular electrical stimulation robot after stroke. Neurorehabil Neural Repair.

[34] Rodgers H, Bosomworth H, Krebs HI, van Wijck F, Howel D, Wilson N, Aird L, Alvarado N, Andole S, Cohen DL (2019). Robot assisted training for the upper limb after stroke (RATULS): a multicentre randomised controlled trial. Lancet..

[35] Mihara M, Hattori N, Hatakenaka M, Yagura H, Kawano T, Hino T, Miyai I (2013). Near-infrared spectroscopy-mediated neurofeedback enhances efficacy of motor imagery-based training in poststroke victims: a pilot study. Stroke..

[36] van Dokkum LEH, Ward T, Laffont I (2015). Brain computer interfaces for neurorehabilitation: its current status as a rehabilitation strategy post-stroke. Ann Phys Rehabil Med.

[37] Ono T, Shindo K, Kawashima K, Ota N, Ito M, Ota T, Mukaino M, Fujiwara T, Kimura A, Liu M (2014). Brain-computer interface with somatosensory feedback improves functional recovery from severe hemiplegia due to chronic stroke. Front Neuroeng.

[38] Nitsche MA, Paulus W (2000). Excitability changes induced in the human motor cortex by weak transcranial direct current stimulation. J Physiol.

[39] Matsumoto J, Fujiwara T, Takahashi O, Liu M, Kimura A, Ushiba J (2010). Modulation of mu rhythm desynchronization during motor imagery by transcranial direct current stimulation. J Neuroeng Rehabil.

[40] Wei P, He W, Zhou Y, Wang L (2013). Performance of motor imagery brain-computer interface based on anodal transcranial direct current stimulation modulation. IEEE Trans Neural Syst Rehabil Eng..

[41] Moher D, Liberati A, Tetzlaff J, Altman DG (2009). Preferred reporting items for systematic reviews and meta-analyses: the PRISMA statement. Ann Intern Med.

[42] Moseley AM, Herbert RD, Sherrington C, Maher CG (2002). Evidence for physiotherapy practice: a survey of the physiotherapy evidence database (PEDro). Aust J Physiother.

[43] Foley NC, Teasell RW, Bhogal SK, Speechley MR (2003). Stroke rehabilitation evidence-based review: methodology. Top Stroke Rehabil.

[44] Chhatbar PY, Ramakrishnan V, Kautz S, George MS, Adams RJ, Feng W (2016). Transcranial direct current stimulation post-stroke upper extremity motor recovery studies exhibit a dose-response relationship. Brain Stimul.

[45] Higgins J, Green S (2011). Cochrane handbook for systematic reviews of interventions version 5.1. 0 (updated March 2011).

[46] Jang YY, Kim TH, Lee BH (2016). Effects of brain–computer interface-controlled functional electrical stimulation training on shoulder subluxation for patients with stroke: a randomized controlled trial. Occup Ther Int.

[47] Rayegani SM, Raeissadat SA, Sedighipour L, Rezazadeh IM, Bahrami MH, Eliaspour D, Khosrawi S (2014). Effect of neurofeedback and electromyographic-biofeedback therapy on improving hand function in stroke patients. Top Stroke Rehabil.

[48] Review Manager (RevMan) [Computer Program] (2014). Version 5.3 Copenhagen: The Nordic Cochrane Centre, The Cochrane Collaboration.

[49] Varkuti B, Guan C, Pan Y, Phua KS, Ang KK, Kuah CWK, Chua K, Ang BT, Birbaumer N, Sitaram R (2013). Resting state changes in functional connectivity correlate with movement recovery for BCI and robot-assisted upper-extremity training after stroke. Neurorehabil Neural Repair.

[50] Ang KK, Guan C, Phua KS, Wang C, Zhou L, Tang KY, Ephraim Joseph GJ, Kuah CW, Chua KS (2014). Brain-computer interface-based robotic end effector system for wrist and hand rehabilitation: results of a three-armed randomized controlled trial for chronic stroke. Front Neuroeng..

[51] Kim T, Kim S, Lee B (2016). Effects of action observational training plus brain-computer interface-based functional electrical stimulation on paretic arm motor recovery in patient with stroke: a randomized controlled trial. Occup Ther Int.

[52] Frolov AA, Mokienko O, Lyukmanov R, Biryukova E, Kotov S, Turbina L, Nadareyshvily G, Bushkova Y (2017). Post-stroke rehabilitation training with a motor-imagery-based brain-computer interface (BCI)-controlled hand exoskeleton: a randomized controlled multicenter trial. Front Neurosci.

[53] Ramos-Murguialday A, Curado MR, Broetz D, Yilmaz Ö, Brasil FL, Liberati G, Garcia-Cossio E, Cho W, Caria A, Cohen LG (2019). Brain-machine interface in chronic stroke: randomized trial long-term follow-up. Neurorehabil Neural Repair.

[54] Ang KK, Guan C, Phua KS, Wang C, Zhao L, Teo WP, Chen C, Ng YS, Chew E (2015). Facilitating effects of transcranial direct current stimulation on motor imagery brain-computer interface with robotic feedback for stroke rehabilitation. Arch Phys Med Rehabil.

[55] Kasashima-Shindo Y, Fujiwara T, Ushiba J, Matsushika Y, Kamatani D, Oto M, Ono T, Nishimoto A, Shindo K, Kawakami M (2015). Brain-computer interface training combined with transcranial direct current stimulation in patients with chronic severe hemiparesis: proof of concept study. J Rehabil Med.

[56] Buch E, Weber C, Cohen LG, Braun C, Dimyan MA, Ard T, Mellinger J, Caria A, Soekadar S, Fourkas A (2008). Think to move: a neuromagnetic brain-computer interface (BCI) system for chronic stroke. Stroke..

[57] Tung SW, Guan C, Ang KK, Phua KS, Wang C, Zhao L, Teo WP, Chew E. Motor imagery BCI for upper limb stroke rehabilitation: an evaluation of the EEG recordings using coherence analysis. Conf Proc IEEE Eng Med Biol Soc. 2013:261–4. 10.1109/EMBC.2013.6609487 24109674

[58] Morone G, Pisotta I, Pichiorri F, Kleih S, Paolucci S, Molinari M, Cincotti F, Kübler A, Mattia D (2015). Proof of principle of a brain-computer interface approach to support poststroke arm rehabilitation in hospitalized patients: design, acceptability, and usability. Arch Phys Med Rehabil.

[59] Kawakami M, Fujiwara T, Ushiba J, Nishimoto A, Abe K, Honaga K, Nishimura A, Mizuno K, Kodama M, Masakado Y (2016). A new therapeutic application of brain-machine interface (BMI) training followed by hybrid assistive neuromuscular dynamic stimulation (HANDS) therapy for patients with severe hemiparetic stroke: a proof of concept study. Restor Neurol Neurosci.

[60] Kotov SV, Turbina LG, Bobrov PD, Frolov AA, Pavlova OG, Kurganskaya ME, Biryukova EV (2016). Rehabilitation of stroke patients with a bioengineered “brain–computer interface with exoskeleton” system. Neurosci Behav Physiol.

[61] Bundy DT, Souders L, Baranyai K, Leonard L, Schalk G, Coker R, Moran DW, Huskey T, Leuthardt EC (2017). Contralesional brain-computer interface control of a powered exoskeleton for motor recovery in chronic stroke survivors. Stroke..

[62] Ibáñez J, Monge-Pereira E, Molina-Rueda F, Serrano JI, Del Castillo MD, Cuesta-Gómez A, Carratalá-Tejada M, Cano-de-la-Cuerda R, Alguacil-Diego IM, Miangolarra-Page JC (2017). Low latency estimation of motor intentions to assist reaching movements along multiple sessions in chronic stroke patients: a feasibility study. Front Neurosci.

[63] Sullivan JL, Bhagat NA, Yozbatiran N, Paranjape R, Losey CG, Grossman RG, Contreras-Vidal JL, Francisco GE, O’Malley MK. Improving robotic stroke rehabilitation by incorporating neural intent detection: preliminary results from a clinical trial. IEEE Int Conf Rehabil Robot. 2017;2017:122–7. 10.1109/ICORR.2017.8009233. 10.1109/ICORR.2017.8009233 PMC6037537 28813805

[64] Nishimoto A, Kawakami M, Fujiwara T, Hiramoto M, Honaga K, Abe K, Mizuno K, Ushiba J, Liu M (2018). Feasibility of task-specific brain-computer interface training for upper-extremity paralysis in patients with chronic hemiparetic stroke. J Rehabil Med.

[65] Chowdhury A, Meena YK, Raza H, Bhushan B, Uttam AK, Pandey N, Hashmi AA, Bajpai A, Dutta A, Prasad G (2018). Active physical practice followed by mental practice using BCI-driven hand exoskeleton: a pilot trial for clinical effectiveness and usability. IEEE J Biomed Health Inform.

[66] Norman SL, McFarland DJ, Miner A, Cramer SC, Wolbrecht ET, Wolpaw JR, Reinkensmeyer DJ. Controlling pre-movement sensorimotor rhythm can improve finger extension after stroke. J Neural Eng. 2018;15. 10.1088/1741-2552/aad724. 10.1088/1741-2552/aad724 PMC6158016 30063219

[67] Remsik AB, Dodd K, Williams L, Thoma J, Jacobson T, Allen JD, Advani H, Mohanty R, McMillan M, Rajan S (2018). Behavioral outcomes following brain-computer interface intervention for upper extremity rehabilitation in stroke: a randomized controlled trial. Front Neurosci.

[68] Tabernig CB, Lopez CA, Carrere LC, Spaich EG, Ballario CH. Neurorehabilitation therapy of patients with severe stroke based on functional electrical stimulation commanded by a brain computer interface. J Rehabil Assist Technol Eng. 2018;5. 10.1177/2055668318789280. 10.1177/2055668318789280 PMC6453036 31191948

[69] Carino-Escobar RI, Carrillo-Mora P, Valdés-Cristerna R, Rodriguez-Barragan MA, Hernandez-Arenas C, Quinzaños-Fresnedo J, Galicia-Alvarado MA, Cantillo-Negrete J. Longitudinal analysis of stroke patients’ brain rhythms during an intervention with a brain-computer interface. Neural Plast. 2019. 10.1155/2019/7084618. 10.1155/2019/7084618 PMC6487113 31110515

[70] Foong R, Ang KK, Quek C, Guan C, Phua KS, Kuah CWK, Deshmukh VA, Yam LHL, Rajeswaran DK, Tang N, et al. Assessment of the efficacy of EEG-based MI-BCI with visual feedback and EEG correlates of mental fatigue for upper-limb stroke rehabilitation. IEEE Trans Biomed Eng. 2019. 10.1109/TBME.2019.2921198. 10.1109/TBME.2019.2921198 31180829

[71] Rathee D, Chowdhury A, Meena YK, Dutta A, McDonough S, Prasad G (2019). Brain-machine interface-driven post-stroke upper-limb functional recovery correlates with beta-band mediated cortical networks. IEEE Trans Neural Syst Rehabil Eng.

[72] Acciarresi M, Bogousslavsky J, Paciaroni M (2014). Post-stroke fatigue: epidemiology, clinical characteristics and treatment. Eur Neurol.

[73] Cohen J (1988). Statistical power analysis for the behavioral sciences.

[74] Frolov AA, Mokienko O, Lyukmanov RKH, Chernikova LA, Kotov SV, Turbina LG, Bobrov PD, Biryukova EV, Kondur AA, Icanova GE (2016). Preliminary results of a controlled study of BCI–exoskeleton technology efficacy in patients with poststroke arm paresis. Bulletin Of RSMU.

[75] Guerra ZF, Lucchetti ALG, Lucchetti G (2017). Motor imagery training after stroke: a systematic review and meta-analysis of randomized controlled trials. J Neurol Phys.

[76] Mehrholz J, Pohl M, Platz T, Kugler J, Elsner B (2018). Electromechanical and robot-assisted arm training for improving activities of daily living, arm function, and arm muscle strength after stroke. Cochrane Database Syst Rev.

[77] Howlett OA, Lannin NA, Ada L, McKinstry C (2015). Functional electrical stimulation improves activity after stroke: a systematic review with meta-analysis. Arch Phys Med Rehabil.

[78] Wu CW, van Gelderen P, Hanakawa T, Yaseen Z, Cohen LG (2005). Enduring representational plasticity after somatosensory stimulation. Neuroimage.

[79] Petrof I, Viaene AN, Sherman SM (2015). Properties of the primary somatosensory cortex projection to the primary motor cortex in the mouse. J Neurophysiol.

[80] Fink AJ, Croce KR, Huang ZJ, Abbott LF, Jessell TM, Azim E (2014). Presynaptic inhibition of spinal sensory feedback ensures smooth movement. Nature..

[81] Asai T (2015). Feedback control of one’s own action: self-other sensory attribution in motor control. Conscious Cogn.

[82] Sitaram R, Ros T, Stoeckel L, Haller S, Scharnowski F, Lewis-Peacock J, Weiskopf N, Blefari ML, Rana M, Oblak E (2016). Closed-loop brain training: the science of neurofeedback. Nat Rev Neurosci.

[83] Daly JJ, Wolpaw JR (2008). Brain–computer interfaces in neurological rehabilitation. Lancet Neurol.

[84] Thibaut A, Chatelle C, Ziegler E, Bruno MA, Laureys S, Gosseries O (2013). Spasticity after stroke: physiology, assessment and treatment. Brain Inj.

[85] Hsieh YW, Wang CH, Wu SC, Chen PC, Sheu CF, Hsieh CL (2007). Establishing the minimal clinically important difference of the Barthel index in stroke patients. Neurorehabil Neural Repair.

[86] Wieloch T, Nikolich K (2006). Mechanisms of neural plasticity following brain injury. Curr Opin Neurobiol.

[87] Pekna M, Pekny M, Nilsson M (2012). Modulation of neural plasticity as a basis for stroke rehabilitation. Stroke..

[88] Wander JD, Blakely T, Miller KJ, Weaver KE, Johnson LA, Olson JD, Fetz EE, Rao RP, Ojemann JG (2013). Distributed cortical adaptation during learning of a brain–computer interface task. Proc Natl Acad Sci U S A.

[89] Halder S, Agorastos D, Veit R, Hammer EM, Lee S, Varkuti B, Bogdan M, Rosenstiel W, Birbaumer N, Kübler A (2011). Neural mechanisms of brain-computer interface control. Neuroimage..

[90] Sugawara K, Onishi H, Yamashiro K, Kirimoto H, Tsubaki A, Suzuki M, Tamaki H, Murakami H, Kameyama S (2013). Activation of the human premotor cortex during motor preparation in visuomotor tasks. Brain Topogr.

[91] Dean PJ, Seiss E, Sterr A (2012). Motor planning in chronic upper-limb hemiparesis: evidence from movement-related potentials. PLoS One.

[92] McDonnell MN, Stinear CM (2017). TMS measures of motor cortex function after stroke: a meta-analysis. Brain Stimul..

[93] Bestmann S, Krakauer JW (2015). The uses and interpretations of the motor-evoked potential for understanding behaviour. Exp Brain Res.

[94] Bembenek JP, Kurczych K, Karli Nski M, Czlonkowska A (2012). The prognostic value of motor-evoked potentials in motor recovery and functional outcome after stroke: a systematic review of the literature. Funct Neurol.

[95] Mrachacz-Kersting N, Jiang N, Stevenson AJ, Niazi IK, Kostic V, Pavlovic A, Radovanovic S, Djuric-Jovicic M, Agosta F, Dremstrup K (2016). Efficient neuroplasticity induction in chronic stroke patients by an associative brain-computer interface. J Neurophysiol.

[96] Pichiorri F, De Vico FF, Cincotti F, Babiloni F, Molinari M, Kleih SC, Neuper C, Kübler A, Mattia D (2011). Sensorimotor rhythm-based brain-computer interface training: the impact on motor cortical responsiveness. J Neural Eng.

[97] Young BM, Nigogosyan Z, Walton LM, Song J, Nair VA, Grogan SW, Tyler ME, Edwards DF, Caldera K, Sattin JA (2014). Changes in functional brain organization and behavioral correlations after rehabilitative therapy using a brain-computer interface. Front Neuroeng..

[98] Xu J, Branscheidt M, Schambra H, Steiner L, Widmer M, Diedrichsen J, Goldsmith J, Lindquist M, Kitago T, Luft AR (2019). Rethinking interhemispheric imbalance as a target for stroke neurorehabilitation. Ann Neurol.

