# Supplementary material for: Immediate and long-term effects of BCI-based rehabilitation of the upper extremity after stroke: a systematic review and meta-analysis
Source: J Neuroeng Rehabil. 2020 Apr 25;17:57. doi: 10.1186/s12984-020-00686-2 (PMC7183617; doi:10.1186/s12984-020-00686-2)
Supplement: Supplementary file 1 — Additional file 1: Figure S1. Funnel plot of the meta-analysis for the immediate effects of BCIs on upper extremity motor function. Figure S2. Funnel plot of the subgroup meta-analysis for the effects of motor imagery based BCIs on upper extremity motor function. Figure S3. Funnel plot of the subgroup meta-analysis for effects of BCIs combined with robots on upper extremity motor function. Figure S4. Funnel plot of the subgroup meta-analysis for the effects of BCIs combined with functional electrical stimulation on upper extremity motor function. Figure S5. Funnel plot of the subgroup meta-analysis for the effects of BCIs combined with visual feedback on upper extremity motor function. Figure S6. Funnel plot of the meta-analysis for the long-term effects of BCIs on upper extremity motor function. [file 12984_2020_686_MOESM1_ESM.docx]

Supplementary section

**Immediate and Long-term effects of BCI-based rehabilitation of the upper extremity after stroke: A Systematic Review and Meta-analysis**

Zhongfei Bai^1,2,3^, Kenneth N. K. Fong^1*^, Jiaqi Zhang^1^, Josephine Chan^4^

^1^Department of Rehabilitation Sciences, The Hong Kong Polytechnic University, Kowloon, Hong Kong SAR

^2^Department of Occupational Therapy, Shanghai YangZhi Rehabilitation Hospital (Shanghai Sunshine Rehabilitation Center), Shanghai, China

^3^Department of Rehabilitation Sciences, Tongji University School of Medicine, Shanghai, China

^4^School of Occupational Therapy, Institute of Health Sciences, Texas Woman’s University, Houston Center, USA





Figure S1. Funnel plot of the meta-analysis for the immediate effects of BCIs on upper extremity motor function





Figure S2. Funnel plot of the subgroup meta-analysis for the effects of motor imagery based BCIs on upper extremity motor function





Figure S3. Funnel plot of the subgroup meta-analysis for effects of BCIs combined with robots on upper extremity motor function





Figure S4. Funnel plot of the subgroup meta-analysis for the effects of BCIs combined with functional electrical stimulation on upper extremity motor function





Figure S5. Funnel plot of the subgroup meta-analysis for the effects of BCIs combined with visual feedback on upper extremity motor function





Figure S6. Funnel plot of the meta-analysis for the long-term effects of BCIs on upper extremity motor function.
